# Testing and Analysis of Selected Navigation Parameters of the GNSS/INS System for USV Path Localization during Inland Hydrographic Surveys

**DOI:** 10.3390/s24082418

**Published:** 2024-04-10

**Authors:** Mariusz Specht

**Affiliations:** Department of Transport and Logistics, Gdynia Maritime University, Morska 81-87, 81-225 Gdynia, Poland; m.specht@wn.umg.edu.pl

**Keywords:** Global Navigation Satellite System (GNSS), Inertial Navigation System (INS), Unmanned Surface Vehicle (USV), path localization, hydrographic surveys

## Abstract

One of the main methods of the path localization of moving objects is positioning using Global Navigation Satellite Systems (GNSSs) in cooperation with Inertial Navigation Systems (INSs). Its basic task is to provide high availability, in particular in areas with limited access to satellite signals such as forests, tunnels or urban areas. The aim of the article is to carry out the testing and analysis of selected navigation parameters (3D position coordinates (Northing, Easting, and height) and Euler angles (pitch and roll)) of the GNSS/INS system for Unmanned Surface Vehicle (USV) path localization during inland hydrographic surveys. The research used the Ellipse-D GNSS/INS system working in the Real Time Kinematic (RTK) mode in order to determine the position of the “HydroDron” Autonomous Surface Vehicle (ASV). Measurements were conducted on four representative routes with a parallel and spiral arrangement of sounding profiles on Lake Kłodno (Poland). Based on the obtained research results, position accuracy measures of the “HydroDron” USV were determined using the Ellipse-D GNSS/INS system. Additionally, it was determined whether USV path localization using a GNSS/INS system working in the RTK mode meets the positioning requirements for inland hydrographic surveys. Research has shown that the Ellipse-D system operating in the RTK mode can be successfully used to position vessels when carrying out inland hydrographic surveys in all International Hydrographic Organization (IHO) Orders (Exclusive, Special, 1a/1b and 2) even when it does not work 100% correctly, e.g., loss of RTK corrections for an extended period of time. In an area with limited coverage of the mobile network operator (30–40% of the time the receiver operated in the differential mode), the positioning accuracy of the “HydroDron” USV using the Ellipse-D GNSS/INS system working in the RTK mode was from 0.877 m to 0.941 m for the R95(2D) measure, depending on the route travelled. Moreover, research has shown that if the Ellipse-D system performed GNSS/INS measurements using the RTK method, the pitch and roll error values amounted to approx. 0.06°, which is almost identical to that recommended by the device manufacturer. However, when working in the differential mode, the pitch and roll error values increased from 0.06° to just over 0.2°.

## 1. Introduction

An Unmanned Surface Vehicle (USV) is a surface vehicle that can move on the water surface without the need of a crew on board [1,2]. USVs are remotely controlled or can operate in autonomous mode, using various sensors, communication and control systems, as well as navigation technologies [3,4]. Unmanned vessels have many advantages such as the ability to carry out long-term missions, work in hard-to-reach places, reduce risk for the crew and the ability to collect large amounts of data [5,6,7] using various sensors such as cameras, echosounders, RAdio Detection AND Ranging (RADAR), Light Detection And Ranging (LiDAR), Inertial Navigation System (INS), Global Navigation Satellite System (GNSS), sonars, etc. [8,9,10,11,12]. Their use is constantly expanding in many areas, and the development of autonomous systems technology enables more and more advanced functions and applications. USVs can be used for a variety of purposes such as archaeological research involving the discovery and examination of sunken ships or plane wrecks [13,14]; biological research enabling the observation of the behaviour of marine animals in their natural environment [15]; oceanographic research to record data on salinity, sea currents, temperature, topography of the sea and ocean floor [16,17]; monitoring the marine environment by measuring water quality, pollution and vegetation levels, as well as the state of marine ecosystems [18,19]; ocean resource exploration such as gas, mines and oil, as well as offshore pipeline/platform construction and maintenance [20,21]; search and rescue of missing people on waterbodies [22,23]; military applications for defence and security purposes such as monitoring coastal areas; protection of offshore installations; or reconnaissance in hard-to-reach places [24,25].

During the implementation of each mission by the USV, it must precisely move along a specific route [26,27]. A commonly applied positioning solution is the simultaneous use (integration) of a GNSS system and an inertial device (INS) [28,29]. A particular advantage of GNSS/INS systems is determining the position of a moving object in the lack of access to satellite signals (forests, tunnels or urban areas) based on a mathematical calculation of the road [30,31]. In order to reduce the position measurement error, inertial and satellite systems are supported by additional devices such as LiDAR, odometry and vision sensors [32,33]. GNSS/INS systems are used primarily in navigation and transport applications, which include mobile phone tracking [34,35]; indoor [36], land [37] and space [38] navigation; geodetic [39,40] and hydrographic [41,42] measurements; driving autonomous ground vehicles [43,44]; as well as car [45,46] and rail transport [47,48].

Cahyadi et al. [49] presents the integration of GNSS and Inertial Measurement Unit (IMU) data for the autonomous navigation of a USV. The research was carried out in the waters of shipyard company in Madura, Indonesia. The IMU and low-cost GNSS receiver were used for registering i-boat USV location data with the use of the Extended Kalman Filter (EKF) to process loosely coupled integration. The research has shown that the GNSS/IMU loosely coupled navigation system enables researchers to determine the position coordinates with an accuracy of 0.3058 m for the Northing and 0.2780 m for the Easting.

Choi et al. [50] proposes a robust localization algorithm for a USV using the Double Deep Q-Network with Action Memory (DDQN-AM). Measurements were conducted in the yacht mooring area at the Korea Maritime and Ocean University (South Korea). The ZED-F9T u-blox Global Positioning System (GPS) receiver and EBIMU-9DOF INS system were used for registering USV location data with the use of the DDQN-AM algorithm. The research has shown that the proposed solution has much better learning performance (accuracy for running time and states) than the DDQN algorithm. Thanks to the use of the DDQN-AM algorithm, USV path localization was higher by 57.6% in comparison to the existing solution, and the mean running time was reduced by 1 min and 30 s.

Liu et al. [51] proposes a novel sensor data fusion algorithm for the autonomous navigation of a USV with the use of the Unscented Kalman Filter (UKF). Measurements were conducted at the Roadford Lake in Devon (England). The electronic compass, GPS receiver and IMU were used for registering Springer USV location data with the use of the conventional UKF and the proposed fuzzy adaptive UKF algorithms. With sufficient a priori knowledge of system noise, the standard UKF algorithm is capable of fusing a variety of raw sensor data and giving precise estimates. The performance of the conventional UKF algorithm is improved using a fuzzy adaptive estimation approach to handle systems without this data. This method enables the algorithm to validate and correct the sensor noise in real time. The results of testing the proposed fuzzy adaptive UKF algorithm in different simulations modelled using real maritime environments are compared to those of the conventional UKF.

The literature research revealed that the integration of GNSS/INS systems is commonly applied for USV path localization. Therefore, the aim of this article is to carry out the testing and analysis of the selected navigation parameters (3D position coordinates (Northing, Easting, and height) and Euler angles (pitch and roll)) of the GNSS/INS system for USV path localization during inland hydrographic surveys.

This publication is structured as follows. Section 2 entitled “Materials and Methods” describes the measurement equipment (“HydroDron” USV and Ellipse-D GNSS/INS system) used during the research, presents how measurements were conducted and the data processing is discussed. Section 3 entitled “Results” shows the obtained research results, including position accuracy measures of the “HydroDron” USV determined using the Ellipse-D GNSS/INS system on four representative routes. Section 4 entitled “Discussion” analyses whether the GNSS/INS system used meets the positioning requirements provided for when carrying out hydrographic surveys for individual International Hydrographic Organization (IHO) orders. The article ends with conclusions summarising the research.

## 2. Materials and Methods

### 2.1. Measurement Equipment

The research used the Ellipse-D GNSS/INS system manufactured by SBG Systems, which consists of the following elements [52]:

- IMU is the main component of an INS. It comprises three gyroscopes, three accelerometers and three magnetometers, which are applied to determine angular velocities and accelerations along the RPY axis: roll, pitch and yaw of the moving vehicle. Detailed technical data of measurement devices included in the Ellipse-D IMU are presented in [52];
- Two GNSS antennas are applied to determine the course of a moving object. They receive satellite signals from GPS: L1 C/A and L2C; GLObal Navigation Satellite System (GLONASS): L1OF and L2OF; BeiDou Navigation Satellite System (BDS): B1 and B2; as well as Galileo: E1 and E5b. The update rate of GNSS signals is 5 Hz;
- sbgCenter software is applied to configure and control GNSS/INS measurement parameters in real time.

The Ellipse-D system includes a Micro-ElectroMechanical System (MEMS)-based IMU and runs an enhanced EKF, which fuses GNSS and inertial data in real-time for increasing the positioning accuracy in harsh environments (urban canyons, tunnels, etc.). Odometer and RTK corrections can be used as aiding inputs to further enhance the navigation solution [53].

The most important functionalities of the Ellipse-D system in the context of hydrographic surveys are the following:

- The GNSS/INS system allows operation in two modes:
  ○ Differential—a method that transmits differential observations via a General Packet Radio Service (GPRS)/Wireless Local Area Network (WLAN), a satellite connection or Very High Frequency (VHF) from a base station (the so-called reference station) with known coordinates to a mobile receiver for which the position coordinates are determined. This measurement technique enables a positioning accuracy of up to tens of centimetres.
  ○ RTK—a method that transmits L1 and L2 carrier phase observations from a base station with known coordinates to a mobile receiver for which the position coordinates are determined. The positioning accuracy at the centimetre level is provided by double-difference measurements and the integer ambiguity resolution.
- GNSS/INS data: accelerations, angles and position coordinates should be recorded as frequently as possible. The Ellipse-D system allows measurements to be registered with the frequency of 200 Hz.

The accuracy characteristics of the Ellipse-D system are presented in Table 1 [54].

Table 1 shows that the Ellipse-D system meets all positioning requirements for all orders of hydrographic surveys if it works in the RTK mode and has access to the GNSS signal. If the satellite signal is lost for approx. 10 s, the INS does not meet the requirements for the most demanding IHO order, i.e., the Exclusive Order, for which the position error should not exceed 1 m (*p* = 0.95). In addition, the Ellipse-D system meets the positioning requirements for selected orders of hydrographic surveys (excluding the Exclusive Order) if it operates in the differential mode and has access to a satellite signal. If the satellite signal is lost for approx. 10 s, the INS does not meet the requirements for the two most demanding IHO orders (Exclusive and Special), for which the position error should not exceed 2 m (*p* = 0.95) [55].

The Ellipse-D system was placed on the “HydroDron” Autonomous Surface Vehicle (ASV), for which it was to be a positioning system for navigation in inland waters. This unmanned vessel is a catamaran with a length of 4 m, a width of 2 m, a height of 1–1.4 m, a draft of 0.5 m and a weight of 300 kg. The measuring speed of the “HydroDron” USV is 3–4 kn., and the max speed is approx. 14 kn. The unmanned vessel allows for the mission to be carried out in four modes:

- Adaptive—designed to move longer distances without operator intervention. After setting the course, the vessel will move it regardless of weather conditions, making corrections on an ongoing basis to maintain the set direction;
- Autonomous—the basic setting allowing autonomous measurements. It enables the setting of route points along which the vessel will move, while simultaneously conducting data acquisition;
- Dynamic positioning—a useful mode when it is necessary to maintain a constant position, e.g., when determining the speed of sound in water in a vertical profile. The vessel will compensate for the impact of wind and other forces acting on it to stop drifting and stay in one place as much as possible;
- Manual—a mode enabling manual control by the operator, e.g., when mooring the vessel or to reach the measurement area with the anti-collision module still active, which will prevent a collision with another watercraft. Additionally, the position, course and all important navigational information are constantly displayed on the chart.

**Figure 1:**
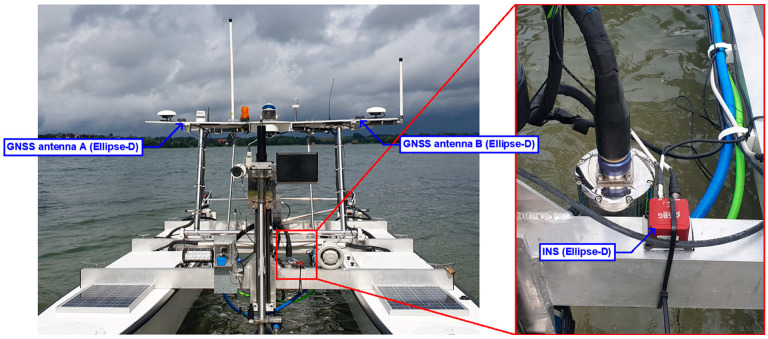
Deployment of the Ellipse-D GNSS/INS system on the “HydroDron” USV.

It should be noted that the “HydroDron” USV is a unique unmanned floating unit in the world that uses multi-sensor data fusion to carry out hydrographic surveys of fixed and floating objects, aids to navigation, features above the vertical reference significant to navigation, coastline, overhead clearances, water flow direction, water flow speed and angular measurements in accordance with the accuracy requirements provided for the most stringent IHO order, i.e., the Exclusive Order. The most important hydrographic and navigation devices installed on the USV include the following: 3DSS-DX-450 interferometric echosounder; SBG Ekinox2-U GNSS/INS system; AML Oceanographic Base X2 Sound Velocity Profiler (SVP) and Conductivity, Temperature, Depth (CTD); Velodyne VLP-16 LiDAR; UMRR-0C Type 42 RADAR, etc. Two GNSS antennas were mounted on the mast of the USV, which were approx. 2 m apart (Figure 1) [4,8,12].

The IMU was mounted near the symmetry axis of the “HydroDron” USV. Other devices, such as the SplitBox computer system and a modem for receiving RTK/Real Time Network (RTN) corrections, were located in one of the hulls of the unmanned vessel. Of course, before starting GNSS/INS measurements, the Ellipse-D system had to be calibrated. It was made using the “HydroDron” USV in the marine mode, which is recommended for marine applications, i.e., those in which there are minor changes in the course of movement and its speed. In accordance with the manufacturer’s recommendations, SBG Systems, the calibration was performed in motion, in such a way as to permanently change the course of the moving object. After 15 min. calibration, the mutual position between the GNSS antennas and the IMU was precisely determined (positioning accuracy: ±1–4 cm and angle accuracy: ±0.04°). With these settings, the GNSS/INS measurements were started.

### 2.2. GNSS/INS Measurements

**Figure 2:**
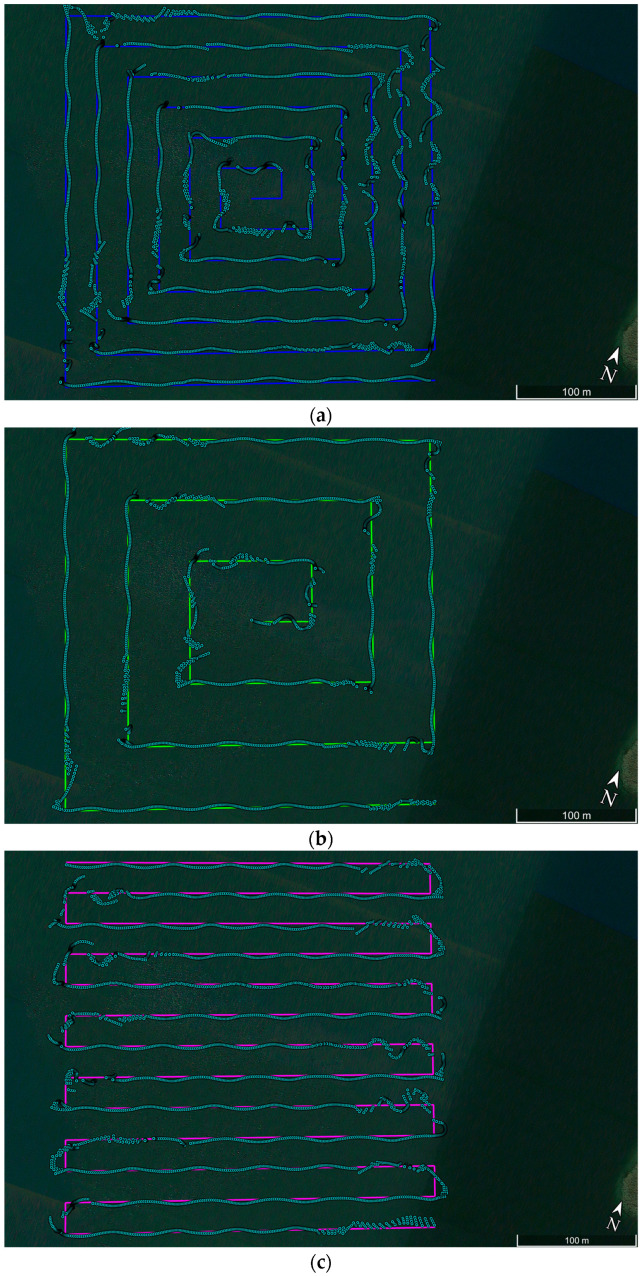

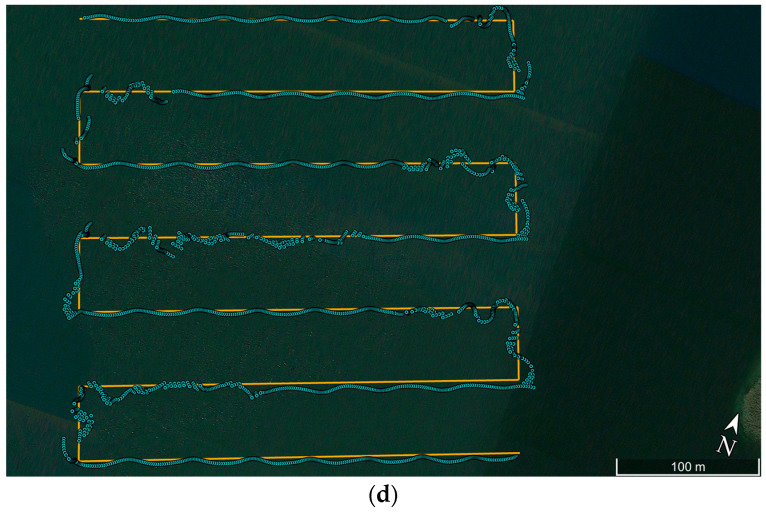
Arrangement of sounding profiles of the routes—spiral every 25 m (**a**) and 50 m (**b**), as well as parallel every 25 m (**c**) and 50 m (**d**), along which the “HydroDron” USV moved. The position coordinates recorded by the Ellipse-D system are marked in sky-blue.

After the installation and calibration of the measurement equipment, GNSS/INS measurements were started in the kinematic mode, which were aimed at determining the positioning accuracy of the “HydroDron” USV using the Ellipse-D system during navigation in inland waters. Lake Kłodno, located in Poland, was chosen as the test waterbody. It was decided to carry out hydrographic surveys in appropriate hydrometeorological conditions, i.e., when there are no waves or sea currents and no wind. GNSS/INS measurements were conducted along four previously designed test routes. The arrangement of the sounding profiles for the first two routes was designed so that the survey lines resembled “tapered squares” (spiral) in the direction of the centre of the test waterbody. For the first and second routes, a constant mutual distance between the sounding profiles was assumed, amounting to 25 m (Figure 2a) and 50 m (Figure 2b), respectively. The arrangement of sounding profiles for the next two routes was designed in such a way that the survey lines were parallel to each other. For the third and fourth routes, similarly to the first two routes, a constant mutual distance between the sounding profiles was assumed, amounting to 25 m (Figure 2c) and 50 m (Figure 2d), respectively. These are typical shapes of routes along which hydrographic surveys are carried out using manned and unmanned vessels [27,56,57,58].

After uploading the designed routes to the autopilot mounted on the drone, the USV began to sail along the set points in autonomous mode. GNSS/INS measurements on the first route (Figure 2a) were carried out between 08:18:16 a.m. and 09:28:46 a.m. Universal Time Coordinated (UTC) on 28 June 2022. During the research, 4231 measurement points were registered with the frequency of 1 Hz. The “HydroDron” USV covered a distance of 5710 m at an average speed amounting to 2.62 kn. The second route (Figure 2b) was travelled between 09:37:40 a.m. and 10:14:58 a.m. UTC on 28 June 2022. During that time, 2239 measurement points were registered with the frequency of 1 Hz. The USV covered a distance of 3115 m with an average speed of 2.70 kn. GNSS/INS measurements on the third route (Figure 2c) were carried out between 10:21:32 a.m. and 11:23:40 a.m. UTC on 28 June 2022. During the research, 3729 measurement points were registered with the frequency of 1 Hz. The “HydroDron” USV covered a distance of 5380 m at an average speed amounting to 2.80 kn. The fourth route (Figure 2d) was travelled between 11:33:40 a.m. and 12:44:45 p.m. UTC on 28 June 2022. During that time, 2466 measurement points were registered with the frequency of 1 Hz. The USV covered a distance of 3516 m with an average speed of 2.77 kn.

### 2.3. GNSS/INS Data Processing

GNSS/INS measurements were carried out in the kinematic mode with the use of the Ellipse-D system, which was mounted on the “HydroDron” USV, on four representative routes. During the tests, the following measurement data were recorded: point number, measurement time, ellipsoidal coordinates, Euler angles, heading, 1D errors and angle measurement errors. They were saved using the sbgCenter software as text files. The GNSS/INS system recorded measurement data using the RTK method with the frequency of 1 Hz. Each of the routes travelled by the “HydroDron” USV was subjected to identical statistical analysis in order to formulate general conclusions. In order to calculate the position accuracy measures (Root Mean Square (RMS), Distance Root Mean Square (DRMS), 2DRMS, Circular Error Probable (CEP), Spherical Error Probable (SEP), R68 and R95) of the USV, the standard deviations of the 1D errors that were recorded during the tests were used (Table 2). For objects moving in real time, the most important position accuracy measures are 2DRMS(2D) and R95(2D), which are characterised by a high level of confidence (95.4–98.2%) [46].

The research also assessed the possibility of using the RTK system to position the USV while conducting hydrographic surveys. This was possible thanks to the use of a mathematical model developed by Specht M. [59], which allows for the determination of whether a given positioning system meets (or not) the requirements provided for in the IHO S-44 standard [55]. The model is based on the theory of the reliability of renewal (repair) processes, where the failure and operation statistics of the system are related to failure and operation times with an exponential distribution. Using this model, a time-dependent availability function (*A*_exp_(*t*)) was calculated for the permissible 2D position error corresponding to a given IHO order [60]:

(1) $$A_{\exp}\left(t\right)=\frac{\mu }{\lambda +\mu }+\frac{\lambda }{\lambda +\mu }\cdot e^{-\left(\lambda +\mu \right)\cdot t},$$

where

*λ*—failure rate,
*μ*—renewal rate.

## 3. Results

Table 3 shows the results of the positioning accuracy of the “HydroDron” USV, which were obtained with the use of the Ellipse-D system in the RTK mode on routes no. 1–4.

From Table 3, it can be seen that the values of individual position accuracy measures of the USV on all routes are close to each other and differ by only a few centimetres. For example, the R68(2D) measures were 0.164 m (route no. 1), 0.113 m (route no. 2), 0.151 m (route no. 3) and 0.203 m (route no. 4). The R95(2D) measures were 0.877 m (route no. 1), 0.886 m (route no. 2), 0.901 m (route no. 3) and 0.941 m (route no. 4). Due to the fact that the values of position accuracy measures obtained by the Ellipse-D system differ from those recommended by the equipment manufacturer using RTK corrections, it was decided to further analyse the values of 1D, 2D and 3D position errors along individual routes.

**Figure 3:**
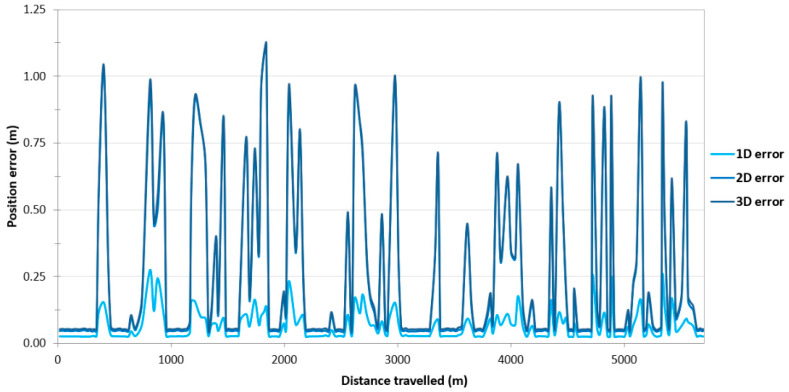
Variability in position errors obtained by the Ellipse-D system in the kinematic mode on the route no. 1.

On the route no. 1, a large variability in the position error values can be observed throughout the entire route (Figure 3). They fall into the following ranges: 0.025–0.365 m (1D error), 0.044–1.118 m (2D position error) and 0.052–1.144 m (3D position error). The cause of this condition is probably the constant loss of reception of RTK corrections due to the Ellipse-D system losing coverage of the Plus mobile network at the place where the measurements were taken.

**Figure 4:**
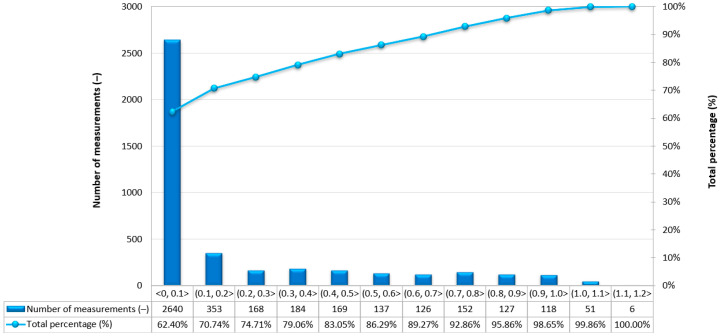
Number of 2D position error intervals obtained by the Ellipse-D system in the kinematic mode on the route no. 1.

Due to the very high variability in the 2D position error, which allows for the determination of the 2D position coordinates of the USV, it was decided to assess what part of the measurements falls within a given error range. For the purposes of the research, it was assumed that 2D position errors not exceeding 10 cm would be treated as those recorded using the RTK method. In the event of a loss of coverage by the mobile network operator, the GNSS/INS system switched to the differential mode. Based on Figure 4, it can be concluded that 62.40% of measurements (2640 fixes) were determined using the RTK method, while the remaining 37.60% of measurements were recorded in the differential mode (1591 fixes). The 2D position error of the USV did not exceed 1 m for 98.65% of GNSS/INS measurements.

**Figure 5:**
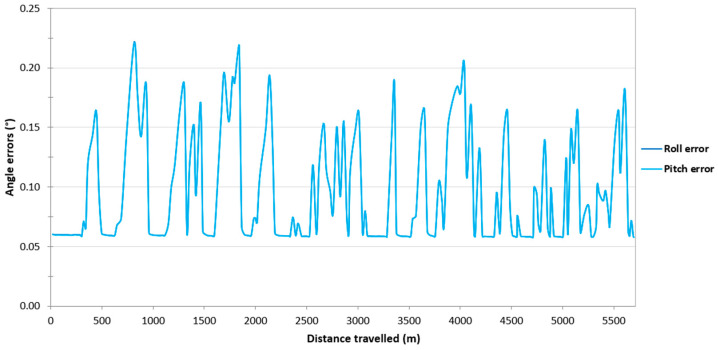
Variability in the pitch and roll errors obtained by the Ellipse-D system in the kinematic mode on the route no. 1.

Then, it was decided to assess the impact of the loss of RTK corrections in the Ellipse-D system on the accuracy of determining the orientation angles of the USV. Based on Figure 5, it can be seen that if the Ellipse-D system performs GNSS/INS measurements using the RTK method, the pitch and roll error values amounted to approx. 0.06°, which is almost identical to that recommended by SBG Systems (Root Mean Square Error (RMSE) of a pitch or roll is 0.05°). However, when working in the differential mode, the pitch and roll error values increase from 0.06° to just over 0.2°. These error values are also close to those recommended by SBG (RMSE of a pitch or roll is 0.1°). Moreover, it should be noted that the pitch and roll error values are almost identical on the route no. 1.

**Figure 6:**
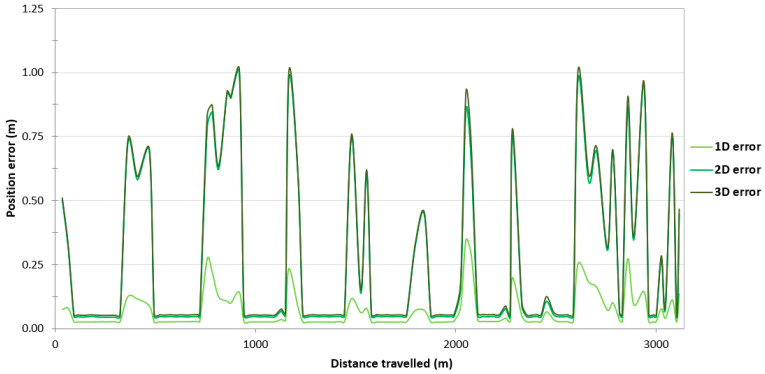
Variability in position errors obtained by the Ellipse-D system in the kinematic mode on the route no. 2.

**Figure 7:**
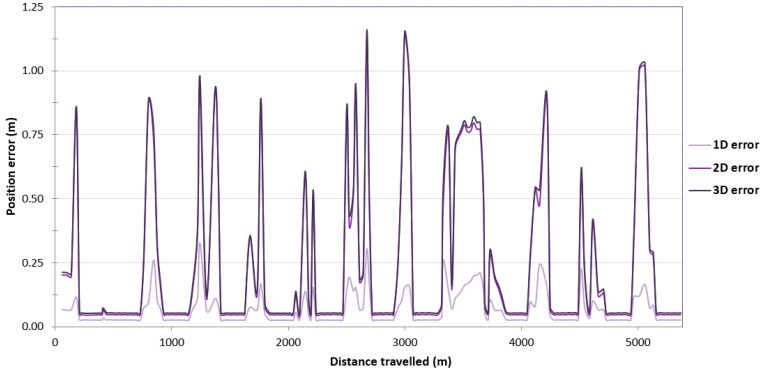
Variability in position errors obtained by the Ellipse-D system in the kinematic mode on the route no. 3.

**Figure 8:**
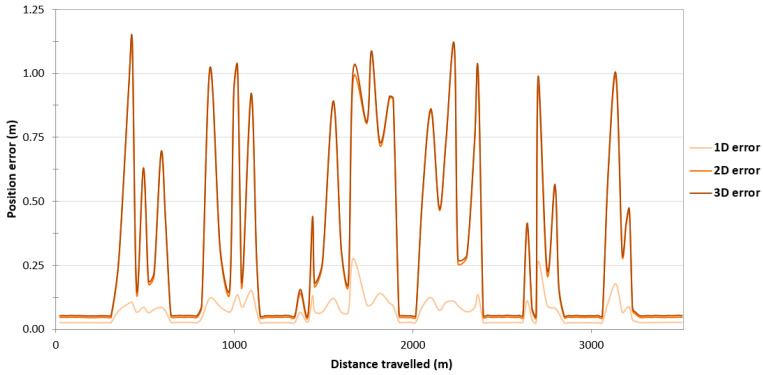
Variability in position errors obtained by the Ellipse-D system in the kinematic mode on the route no. 4.

To assess the reliability of results obtained during GNSS/INS measurements on the route no. 1, the survey results were analysed on the remaining routes. As to the route no. 1, a large variability in the position error values can be observed along routes no. 2–4. On the route no. 2, they fall into the following ranges: 0.031–0.812 m (1D error), 0.044–1.148 m (2D position error) and 0.051–1.157 m (3D position error) (Figure 6). On the route no. 3, the error limits are 0.031–0.810 m (1D error), 0.044–1.145 m (2D position error) and 0.051–1.189 m (3D position error) (Figure 7). On the route no. 4, they fall into the following ranges: 0.031–0.812 m (1D error), 0.044–1.148 m (2D position error) and 0.052–1.154 m (3D position error) (Figure 8). The cause of this condition is probably the constant loss of reception of RTK corrections due to the Ellipse-D system losing coverage of the Plus mobile network at the place where the measurements were taken.

**Figure 9:**
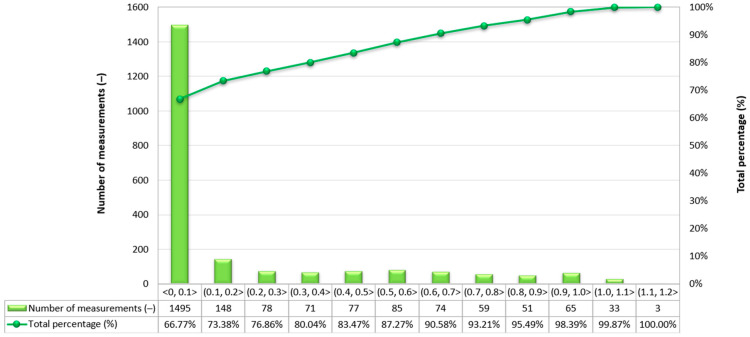
Number of 2D position error intervals obtained by the Ellipse-D system in the kinematic mode on the route no. 2.

**Figure 10:**
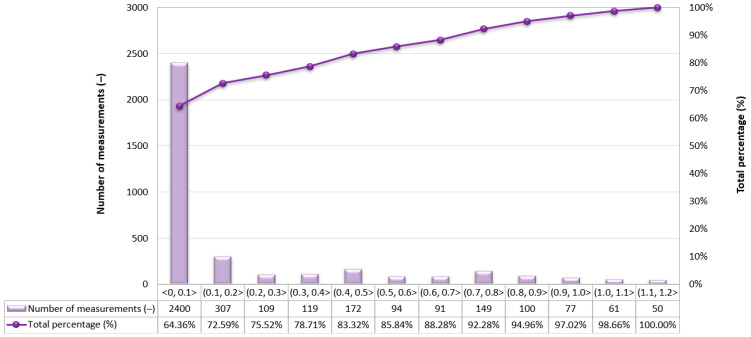
Number of 2D position error intervals obtained by the Ellipse-D system in the kinematic mode on the route no. 3.

**Figure 11:**
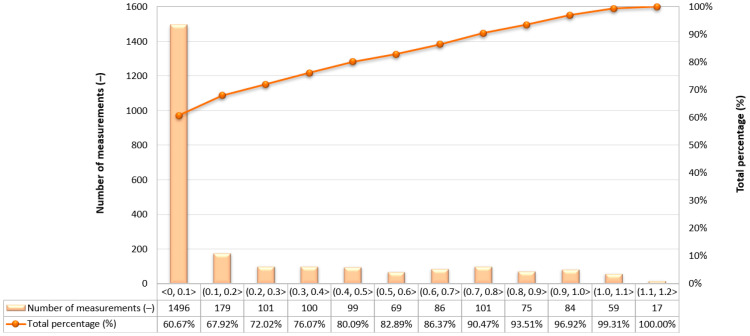
Number of 2D position error intervals obtained by the Ellipse-D system in the kinematic mode on the route no. 4.

Due to the very high variability in the 2D error value on routes no. 2–4, it was decided to assess what part of the measurements falls within a given error range. As to the route no. 1, similar percentages of GNSS/INS measurements using the RTK method were recorded: 66.77% (1495 fixes) on the route no. 2 (Figure 9), 64.36% (2400 fixes) on the route no. 3 (Figure 10) and 60.67% (1496 fixes) on the route no. 4 (Figure 11). The remaining part of the measurements on the analysed routes were recorded in the differential mode. The 2D position error of the USV did not exceed 1 m for nearly 100% of GNSS/INS measurements on routes no. 2 (99.87%), 3 (98.66%) and 4 (99.31%).

**Figure 12:**
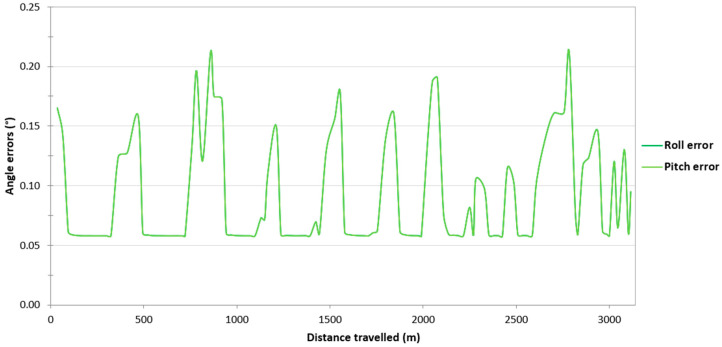
Variability in the pitch and roll errors obtained by the Ellipse-D system in the kinematic mode on the route no. 2.

**Figure 13:**
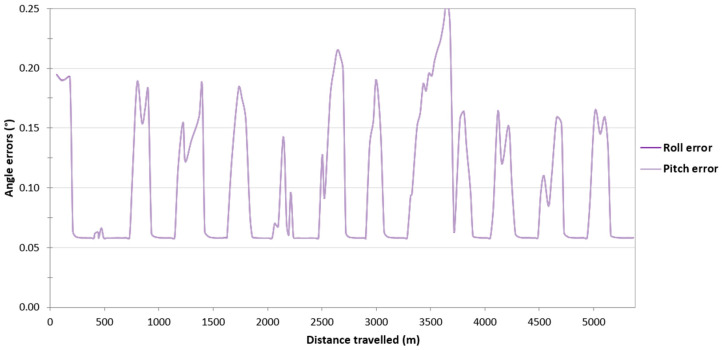
Variability in the pitch and roll errors obtained by the Ellipse-D system in the kinematic mode on the route no. 3.

**Figure 14:**
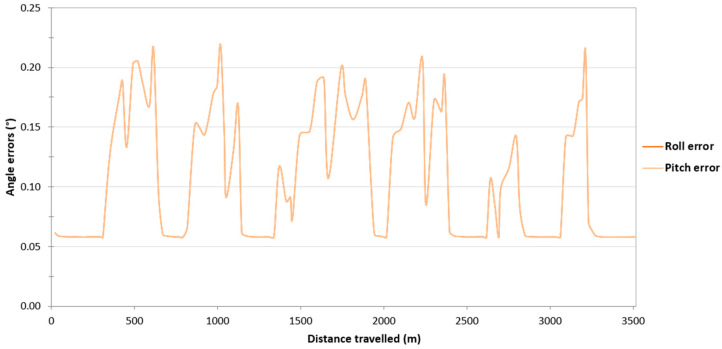
Variability in the pitch and roll errors obtained by the Ellipse-D system in the kinematic mode on the route no. 4.

Then, it was decided to assess the impact of the loss of RTK corrections in the Ellipse-D system on the accuracy of determining the orientation angles of the USV. On routes no. 2–4, similarly to the route no. 1, it can be seen that if the Ellipse-D system performs GNSS/INS measurements using the RTK method, the pitch and roll error values amounted to approx. 0.06°. However, when working in the differential mode, the pitch and roll error values increase from 0.06° to just over 0.2°. Moreover, it should be noted that the pitch and roll error values are almost identical on routes no. 2–4 (Figure 12, Figure 13 and Figure 14).

Next, the mathematical model mentioned in Section 2.3 was used. Calculations and statistical analyses were performed for measurement data recorded by the Ellipse-D system. For each order of hydrographic surveys, characterised by other permitted position errors, the value of the positioning availability function was calculated (Table 4).

## 4. Discussion

The conducted research proves that the Ellipse-D system operating in the RTK mode in an area with limited coverage of the mobile network operator (depending on the route for 60–70% of the time) allows the system to meet the positioning requirements for all five IHO orders: Exclusive, Special, 1a/1b and 2. The availability factor values for the Special, 1a/1b and 2 orders was 100% each time. However, in the case of the Exclusive Order, the availability factor value exceeded the required confidence level of 95% and amounted to 98.63% (route no. 1), 98.39% (route no. 2), 96.99% (route no. 3) and 96.86% (route no. 4).

Identical tests were carried out on routes no. 1 and 2 using two inertial navigation systems, Ekinox2-U and Ellipse-D manufactured by SBG Systems, which were supported by Differential Global Positioning System (DGPS) and RTK receivers, respectively [54]. The Ellipse-D system recording measurement data using the DGPS method obtained higher position error values of the USV than in the kinematic method. For example, the R95(2D) measure was 1.036 m for the route no. 1 and 1.065 m for the route no. 2. In addition, the Ellipse-D system operating in the differential mode did not achieve the required positioning availability for the IHO Special Order: 38.48% for the route no. 1 and 15.54% for the route no. 2.

Similar research was also conducted by Oguntuase et al. [61]. In their research, the authors of the article used the Ellipse-D GNSS/INS system for bathymetric measurements. The aim of this publication was to answer the following questions: what positioning accuracy is provided by the tested GNSS/INS systems and for which IHO orders they can be used. Bathymetric measurements were carried out at the Port of Gulfport, Mississippi, in the USA using a manned survey boat (RV LeMoyne) on which the tested GNSS/INS systems were mounted. The research was carried out in the dynamic mode with an average speed of 2.7 kn. along 42 sounding profiles of 250 m long each. Tests have shown that the mean roll offset between the reference (Applanix POSMV) and tested (SBG Ellipse-D) GNSS/INS systems is 0.005°, and the corresponding 95% ordered statistics are 0.012°. The mean pitch offset between the POSMV and Ellipse-D GNSS/INS systems is 0.233°, and the corresponding 95% ordered statistics are 0.247°. Moreover, the mean height offset between the POSMV and Ellipse-D GNSS/INS systems is 0.048 m, and the corresponding 95% ordered statistics are 0.082 m. Research has shown the Ellipse-D system can meet the accuracy requirements for determining depth in shallow waters (<25 m) provided for the IHO Order 1 using a MultiBeam EchoSounder (MBES) with a 65-degree swath. However, it does not meet the accuracy requirements provided for the most stringent IHO orders (Exclusive and Special).

## 5. Conclusions

Research has shown that the Ellipse-D system operating in the kinematic mode can be successfully used to position vessels such as the “HydroDron” USV, when carrying out hydrographic surveys in all IHO orders (Exclusive, Special, 1a/1b and 2), even when it does not work 100% correctly, e.g., loss of reception of RTK corrections for a long period of time. Determining whether a given positioning system meets the accuracy requirements for individual orders of hydrographic surveys was possible thanks to the use of a mathematical model developed by Specht M. [59].

In an area with limited coverage of the mobile network operator (30–40% of the time the receiver operated in the differential mode), the positioning accuracy of the “HydroDron” USV with the use of the Ellipse-D system operating in the kinematic mode was as follows for the R95(2D) measure: 0.877 m (route no. 1), 0.886 m (route no. 2), 0.901 m (route no. 3) and 0.941 m (route no. 4). If there was no loss of reception of RTK corrections, the positioning accuracy of the vessel would be expected to be several centimetres (*p* = 0.95). It is worth noting that the values of individual position accuracy measures of the USV on all routes are similar and differ by only a few centimetres.

The study also assessed the impact of the loss of RTK corrections in the Ellipse-D system on the accuracy of determining the orientation angles of the USV. If the Ellipse-D system performed GNSS/INS measurements using the RTK method, the pitch and roll error values amounted to approx. 0.06°, which is almost identical to that recommended by SBG Systems (RMSE of a pitch or roll is 0.05°). However, when working in the differential mode, the pitch and roll error values increased from 0.06° to just over 0.2°. These error values are also close to those recommended by SBG Systems (RMSE of a pitch or roll is 0.1°).

In future research, it is planned to use the remaining three main mobile networks in Poland (Orange, Play and T-Mobile) to transmit RTK corrections during hydrographic surveys on Lake Kłodno. Thanks to this, it will be possible to perform a comparative analysis of the obtained values of position accuracy measures of the USV for the four most important mobile operators operating in Poland (Orange, Play, Plus and T-Mobile) and then select the best one in terms of the accuracy of positioning the vessel during the implementation of inland hydrographic surveys. Moreover, it would be worth determining the impact of hydrometeorological conditions on the positioning accuracy of the USV when performing hydrographic surveys in various waterbodies (inland and marine).

## Figures and Tables

**Table 1:** Accuracy characteristics of the Ellipse-D system in the case of access and lack of access to the GNSS signal [54].

RMSE	Time That Has Elapsed Since the GNSS Signal Was Not Available			
	0 s		10 s	
	DGPS	RTK	DGPS	RTK
2D position (m)	1.2	0.01	3	1
Height (m)	1.5	0.02	3.5	1
Pitch, roll (°)	0.1	0.05	0.1	0.05
Course (°)	0.8	0.2	0.8	0.2

**Table 2:** Selected position accuracy measures. Own study based on [46].

Position Accuracy Measure	Dimension	Probability	Definition
RMS	1D	68.3%	The standard deviation of the position coordinate relative to the latitude (*φ*), longitude (*λ*) or height (*h*).
DRMS	2D	63.2–68.3%	The square root calculated from the sum of squared standard deviations of position coordinates relative to *φ*, *λ*, (*h*).
	3D		
2DRMS	2D	95.4–98.2%	Twice the DRMS.
	3D		
CEP	2D	50%	The radius of the circle centred at the true position, containing the position estimate with a confidence level of 50%.
SEP	3D	50%	The radius of the sphere centred at the true position, containing the position estimate with a confidence level of 50%.
R68	2D	68%	The radius of the circle (sphere) centred at the true position, containing the position estimate with a confidence level of 68%.
	3D		
R95	2D	95%	The radius of the circle (sphere) centred at the true position, containing the position estimate with a confidence level of 95%.
	3D		

**Table 3:** Positioning accuracy of the “HydroDron” USV with the use of the Ellipse-D system in the RTK mode on routes no. 1–4.

Statistics of the Position Error	Route No. 1	Route No. 2	Route No. 3	Route No. 4
Number of measurements	4231	2239	3729	2466
RMS(*ϕ*)	0.249 m	0.241 m	0.258 m	0.276 m
RMS(*λ*)	0.249 m	0.241 m	0.258 m	0.276 m
RMS(*h*)	0.081 m	0.087 m	0.089 m	0.075 m
DRMS(2D)	0.352 m	0.341 m	0.364 m	0.390 m
2DRMS(2D)	0.705 m	0.683 m	0.729 m	0.781 m
DRMS(3D)	0.362 m	0.352 m	0.375 m	0.398 m
CEP(2D)	0.049 m	0.048 m	0.048 m	0.047 m
R68(2D)	0.164 m	0.113 m	0.151 m	0.203 m
R95(2D)	0.877 m	0.886 m	0.901 m	0.941 m
SEP(3D)	0.056 m	0.054 m	0.054 m	0.054 m
R68(3D)	0.179 m	0.130 m	0.166 m	0.220 m
R95(3D)	0.895 m	0.914 m	0.919 m	0.953 m

**Table 4:** Positioning availability limits determined for the Ellipse-D system in relation to min. positioning requirements required for five IHO orders on routes no. 1–4.

Route Number	Positioning Availability (%)			
	Exclusive Order	Special Order	1a/1b Orders	Order 2
1	98.63	100	100	100
2	98.39	100	100	100
3	96.99	100	100	100
4	96.86	1000	100	100

## Data Availability

The data that has been used is confidential.
